# A plasmonic refractive index sensor with an ultrabroad dynamic sensing range

**DOI:** 10.1038/s41598-019-41353-4

**Published:** 2019-03-26

**Authors:** Yu-Chieh Cheng, Ya-Ju Chang, Yu-Ching Chuang, Bo-Zhi Huang, Chii-Chang Chen

**Affiliations:** 1 Department of Electro-Optical Engineering, National Taipei University of Technology, 10608 Taipei, Taiwan; 2 Department of Optics and Photonics, National Central University, 32001 Jhongli, Taiwan

## Abstract

Refractive index sensors based on surface plasmon resonance (SPR) promise to deliver high sensitivities. However, these sensitivities depend on the derivative of the monitored SPR parameters near resonance, so this dependency leads to a relatively narrow detection range for refractive index changes. Herein, we introduce an idea to improve the detection range refractive index through a high-contrast-index curved waveguide surrounded with an outer gold ring. The proposed detection technique, based on the output power measurement of the curved waveguide, offers a linear response over an ultrabroad range of the refractive index for a surrounding medium from *n* = 1 to 2.36. Meanwhile, an theoretically ultrahigh refractive index resolution (RIU) of 4.53 × 10^−10^ could be accessible for such a broad testing range, available for both gas and aqueous chemical sample refractive indices Furthermore, the power detection approach enables an integrated photodetector for a lab-on-chip sensor platform, revealing a high potential for a multifunctional, compact, and highly sensitive sensor-on-chip device.

## Introduction

Surface plasmon resonance (SPR)^1^ for biosensors has been attracting increasing attention because of its highly enhanced local electromagnetic field and susceptibility to the surrounding medium. After the first use of an SPR sensor for gas detection was realized^2^, the operation of prisms, via the so-called Otto^3^ and Kretschmann configurations^4^, was studied for measurements of physical, chemical and biological quantities, mainly determined by a variance in the refractive index (RI) of an analyte^5^. However, these conventional prism-based SPR sensors are very bulky due to their many optical and mechanical components.

**Figure 1:**
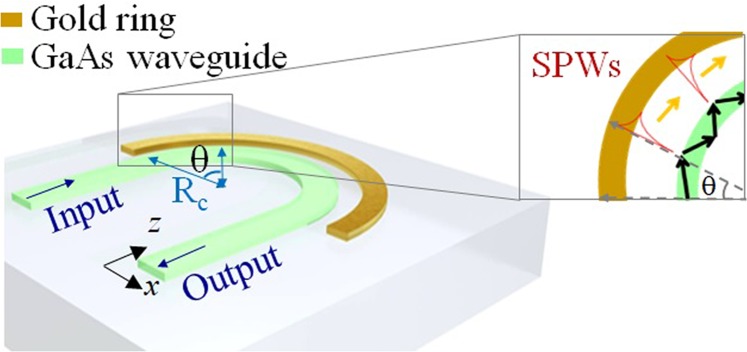
Schematic of a curved-waveguide-based SPR sensor. The configuration contains a curved GaAs waveguide surrounding by an outer gold ring. The inset picture shows multiple SPW excitations along the surface of the gold ring via ATR.

Many improved and miniatured SPR sensors have been accomplished using optical fibers^6–8^, varying microcavities coupled with metal-insulator-metal (MIM) waveguides^9,10^, Mach-Zehnder interferometric biosensor^11^, metallic nanoparticles^12–14^ or plasmonic metamaterials^15–17^. However, these state-of-the-art SPR RI sensors with promising sensing performance determined by the derivative of the monitored SPR parameters (e.g., resonant angular, resonant wavelength, intensity near the resonance) are only operated under a narrow detection range for RI changes. Another critical limitation of these SPR sensors is based on the measurement of the resonance of an optical wave being restricted to the use of a costly and bulky spectrometer, which is not achievable for an on-chip device. Therefore, developing a compact SPR sensor with higher sensitivities for a broader dynamic sensing range has been challenging. In this work, we numerically demonstrate an ultrabroad detection variance in a SPR sensor based on a curved high-index waveguide. An air gap is introduced between the curved waveguide and a gold ring, similar to the Otto configuration illustrated in Fig. 1. Part of the guided energy is transferred to excited surface plasma waves (SPWs) by attenuated total reflection (ATR). Due to high losses in a metallic medium at the near-infrared spectral regions, excited multiple SPWs result in considerable energy losses along the curved waveguide. The RI change causes more attenuation, which introduces stronger SPWs. As a result, the output power of the curved waveguide depends on the RI change, which is the main working principle leveraged in this study.

## Results and Discussions

**Figure 2:**
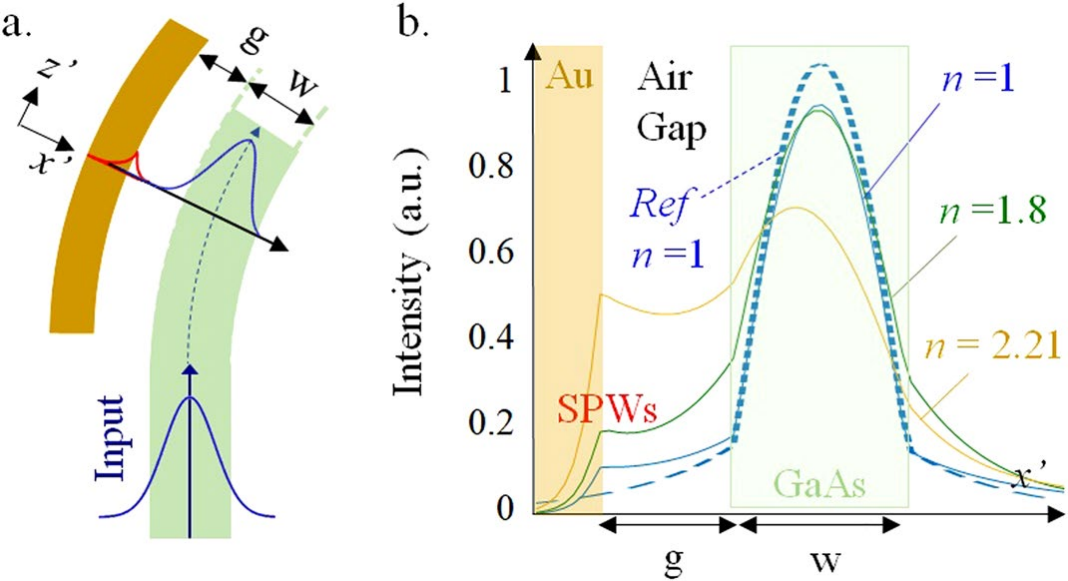
Field intensity distributions of a TM mode propagation at the second SPW excitation. (**a**) An illustration of multiple SPW excitations along the surface of the gold ring. (**b**) The blue, green and yellow lines represent the TM field intensity distributions for three background indices *n* = 1, *n* = 1.8 and *n* = 2.21, respectively. The dashed blue line represents the field distribution for a curved waveguide without a gold ring.

The primary design of a high-index GaAs curved waveguide with *n*_GaAS_ = 3.4 is capable of strong light confinement over a wide RI range. A width and a radius of curvature for the curved waveguide were determined by a conformal transformation^18^ and the Helmholtz equation to optimize bending losses^19^. The optimal width and the radius of the curved single mode waveguide are w = 0.25 μm and Rc = 5 μm. The analytical results were also verified by using a two-dimensional (2D) finite-difference time-domain (FDTD) method and the FDTD results shows that, for TM polarization, the normalized output power of the designed curved waveguide in the background index *n* = 1 is 0.998. The configuration of the waveguide through the dielectric barrier of a narrow air gap of g = 0.2 μm to the metallic ring was essential for an excitation of a SPW, as illustrated in Fig. 2a. The TM fundamental mode of a slab waveguide with a step RI profile was set as the launched source at the operating wavelength of λ = 1.55 μm.

Different field intensity distributions occur for a TM mode propagation with a second SPW excitation for three different background indices of *n* = 1, *n* = 1.8 and *n* = 2.21, which are shown as blue, green and yellow lines in Fig. 2b. The dashed blue line shows the curved waveguide without a gold ring as a reference. Clearly, the energy propagation decreases gradually after introducing a metallic ring due to the multiple SPWs excitations by the ATR. Furthermore, when the RI of the surrounding medium increases, the evanescent field outside the waveguide in the air or the surrounding medium increases, resulting in a stronger SPW. Therefore, the longer evanescent tail outside the waveguide for a higher background index leads to more dissipation. Therefore, the change in the background index, which is one of the essential features in an RI sensor, can be determined by measuring the output power.

**Figure 3:**
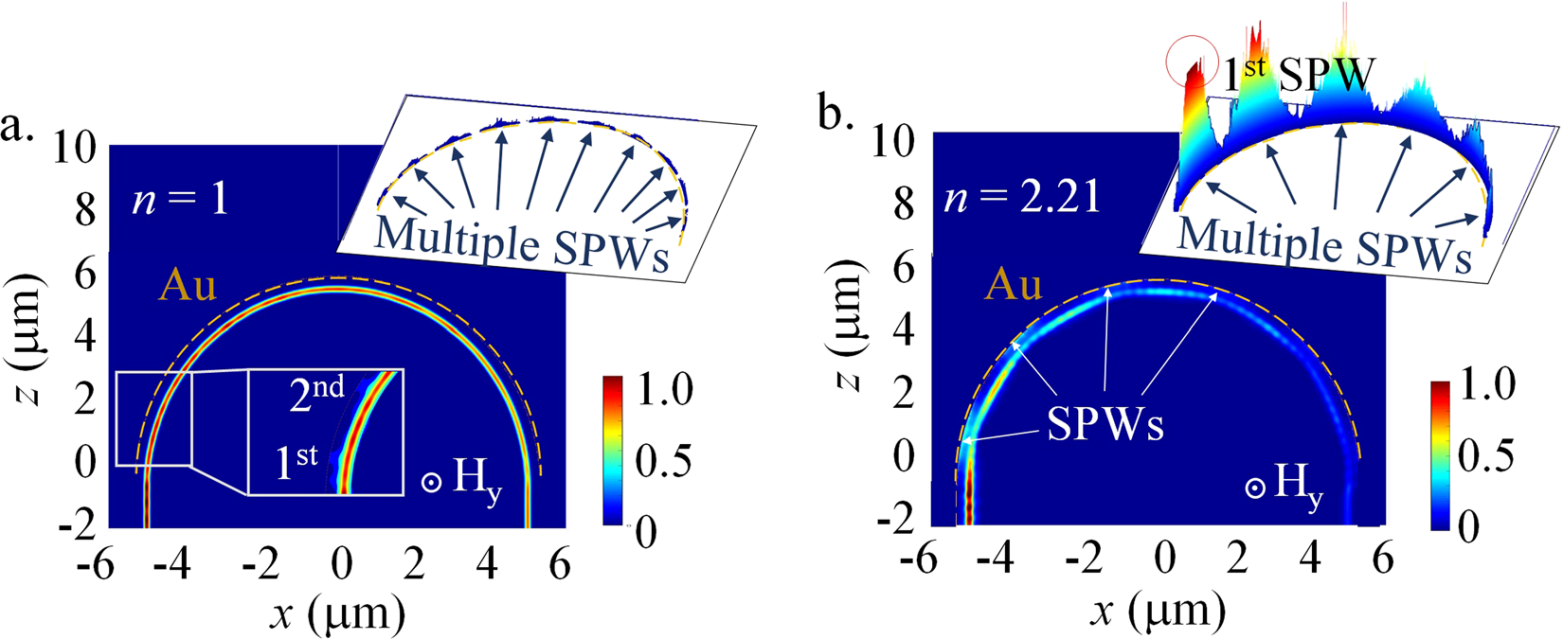
Field intensity distributions of a TM mode propagation. The curved GaAs waveguide with an outer gold ring for different background indices of (**a**) *n* = 1 and (**b**) *n* = 2.21. The upper insert pictures show the intensity distribution along the surfaces of the outer gold ring [orange dashed lines]. The color bar of the insert picture in (**a**) is different than the others to allow observation of the 1^st^ and 2^nd^ SPWs.

In particular, the curved configuration provides multiple SPWs along the gold ring. Unlike other SPR sensors, including cavities structures with a side-coupled MIM waveguide that merely provide the SPW excitations at once^9,10^, the gold ring can excite multiple SPWs along the curved waveguide. Herein, we examine the role of these multiple SPW excitations in the output power losses by studying the two individual curved waveguides at different backgrounds *n* = 1 and *n* = 2.21, as illustrated in Fig. 3a,b. For the background index *n* = 1 and *n* = 2.21, the reduction of the guided energy along the curved waveguide is approximately 21.1% and 87.7%, respectively. The insert pictures in Fig. 3 show their considerable energy dissipation due to multiple SPW excitations.

### The sensing performance

**Figure 4:**
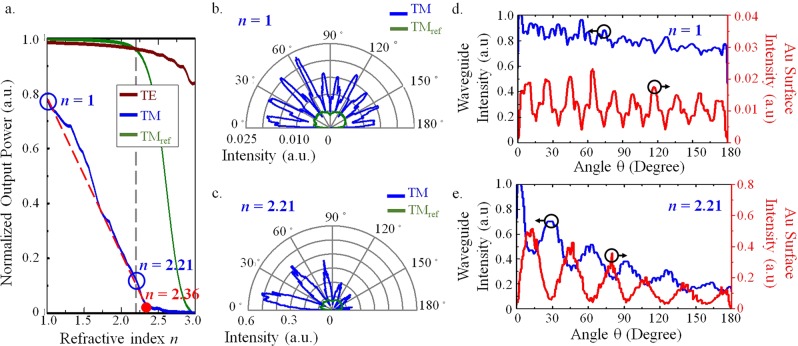
The normalized output power of the curved waveguide and the field distribution along the gold rings varies with background indices. (**a**) The normalized output power as a function of the RI of the background. Three cases are studied: the blue line considers a TM mode launched in the curved waveguide with a metallic ring. The green line corresponds to the same curved waveguide for a TM mode without an outer ring. The brown line represents the same configuration with an outer gold ring, but a TE guided mode is considered. These three field intensity distributions along the surfaces of the outer rings are discussed for two different background indices: (**b**) *n* = 1 and (**c**) *n* = 2.21. The field intensity distributions along the surfaces of the outer rings (as shown with the red color) and along the center of the curved waveguide for two different background indices: (**d**) n = 1 and (**e**) n = 2.21.

The refractive index resolution (RIU) is the key parameter for evaluating the sensing performance of an RI SPR sensor. The definition of the RIU is the minimum detectable RI change. Most SPR sensors have been studied for the enhancement of the spectral sensitivity^20,21^ *s* = *dλ*/RIU. These high-sensitivity SPR sensors provide an ultrahigh resolution RIU that is, however, restricted by the resolution of the spectrometer *dλ*. On the other hand, a real-time RI detecting method based on monitoring the variations in output light power^22^ is considered for our proposed structure. For a higher RI in a surrounding medium, the output power decreases linearly, as shown by the red dashed line in Fig. 4a. Once the guiding condition for a TM mode is unusable for a higher-RI background, those guiding losses introduce more intensity at the first SPW excitation, which result in considerable energy dissipation. Eventually, the output power vanishes for a background RI larger than *n* = 2.36, as depicted by the blue line in Fig. 4a. Therefore, the proposed structure is available for ultrabroad testing due to the high-index-contrast waveguide structure capable of strong light confinement. It is noted that the field intensity distributions vary with the refractive indices as shown in the Supplementary Video.

Our proposed configuration is also compared with two other cases: (1) the same configuration for a TE mode [the brown line in Fig. 4a] and (2) the same waveguide structure without a gold ring for a TM mode [the green line in Fig. 4a]. Due to the absence of the SPWs, these two configurations remain at a high output power. However, our SPR waveguide-based sensor offers strong multiple SPW excitations, as shown by the blue lines in Fig. 4a. The intensity of the guided energy gradually decreases due to the energy dissipation from every SPW excitation, as shown in Fig. 4b,c. Each SPW excitation results in lower propagation energy that induces less energy in the next SPW due to the weaker evanescent field. Such a dynamic sensing interaction Fig. 4d,e due to the SPWs in a sequence leads to a quasi-linear reduction. Impressively, an ultrahigh RIU of 4.53 × 10^−10^ is achievable via a high-resolution photodiode, δ*P*_pd_ = 0.01 nW (see the Methods section for the details).

**Figure 5:**
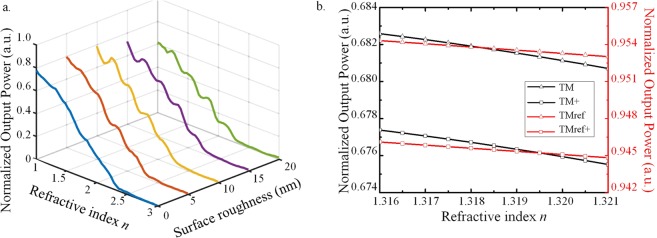
Sensing accuracy dependent on the experimental circumstances. (**a**) The performance of normalized output power versus refractive index for different surface roughness σ which varies from σ = 0 to σ = 20 nm. The effective operating range Δn = 1.36 is divided into several segments due to fluctuations. (**b**) The normalized output power versus refractive index changes with and without the consideration of the optical absorption of water as presented with the square and triangle symbol. The black and red lines present the curved waveguide with/without outer gold rings.

It should be noted that the sensing accuracy is very dependent on the experimental circumstances. For example, a fluctuation of input power should be considered in this proposed structure so the measured output power should be normalized with the fluctuating input power. In Fig. 4a, the normalized output power is calculated by the ratio of the output to input power. It is achievable by using another photodiode to monitor the input fluctuation^23^. Furthermore, the sensing performance may also differ due to fabrication imperfections. Numerical investigations of the surface roughness on the variation of the output power are as shown in Fig. 5a. The linear range of detection range is still broad from n = 1~2.36 with the surface roughness of the root mean square (RMS) value of σ = 20 nm. However, the scattering losses cause the fluctuations of the normalized power variation so the local RIU varies with RI. Figure 5a shows that the proposed curved waveguide considering the surface roughness of σ = 5 nm still numerically implements the linearly broad detection range Δn = 1.36. The fabrication of the curved waveguide with the surface roughness of σ = 5 nm is achievable based on the reported study^24^, which numerically and experimentally reported that the GaAs microresonators have been successfully demonstrated with the surface roughness of σ = 4.6 nm. However, the RIU won’t be constant due to the power fluctuations for the larger surface roughness up to σ = 20 nm. The working ranges are divided into few segments with an estimated individual detecting range about Δn = 0.16 due to the fluctuations. Nevertheless, these curved waveguides are still capable of the customization for sensing local RI segments within the broad range from n = 1 to n = 2.36.

It should also be noted that waveguide-type sensors that measure output power could hardly distinguish the power losses from optical absorption or the varying refractive index of a specimen. Nevertheless, the RI sensor based on power measurement is also applicable for common specimens such as methane gas or NaCl because they are nonabsorptive at λ = 1.55 μm. Moreover, the optical absorption of water is also negligible at λ = 1.55 μm in our configuration because of the narrow operating area of SPWs in the air gap. For a potential application for aqueous solution, we have simulated that the normalized power with a consideration of an aqueous solution with the extinction coefficient k = 10^−4^ at λ = 1.55 μm as shown in Fig. 5b. The water absorption between the curved waveguide with and without outer gold rings is around 0.86% and 0.76%. Such the little power difference explains that the water absorption caused by the SPW excitations is not the main issue and it can be compensated by raising the input power. Therefore, the water absorption for our proposed device is irrelevant at the RI measurement.

One of the most essential sensing parameters for an SPR sensor is the operating range of its RI, which is a measurable range of the determined parameter. Although a broader dynamic sensing range for a conventional SPR sensor has been accessible via multiple resonance dips^9^, a dynamic sensing range was only achieved up to ∆*n* = 0.4. However, our SPR sensor numerically presents a quasi-linear response for a broad dynamic sensing range with RI of ∆*n* = 1.36, which can offer solutions for both aqueous and gaseous media. We believe that although the customization of the curved waveguide for certain applications is required, there still is high potential due to the broad detecting range that is not achievable for most presented studies. The SPR sensor based on power measurement presents a quick response in RI changes rather than identifies or characterizes an analyte. For material identifications, an adsorption layer is required to detect a specific gas but, simultaneously, such a technique could demand long detecting time to reach a saturation^25^, not considered in our current study.

## Conclusion

In summary, we numerically demonstrated the ultrabroad dynamic sensing range of an SPR curved-waveguide-based sensor. Similar to the Otto configuration, a sandwich structure with an air gap between the curved waveguide and a gold ring excites multiple SPWs on the surface of the gold ring. The key feature is that the variance of the normalized output power has a linear response to the RI change of the background. Not only a high RIU of 4.53 × 10^−10^ but also the ultrabroad detecting range of the RI of the background from *n* = 1 to *n* = 2.36 is accomplished in the proposed design. Such a significant dynamic sensing range is applicable for both gas and aqueous samples. Impressively, the RIU for gas sensing (higher index-contrast) is the same as for liquid sensing (lower index-contrast). Unlike those conventional SPR sensors utilizing derivations in the SPR dips, the detection approach based on measuring the normalized output power can be accomplished by an on-chip photodiode, which has high potential for the development of portable, compact, and cost-effective on-chip sensing devices.

## Methods

The calculation of the RIU is based on the normalized output power corresponding to the RI changing in the background. In this work, the parameters of the considered photodiode (PD300-IR, Ophir Co., Israel) have a resolution of δ*P*_*pd*_ = 0.01 nW and a maximum measurable power ∆*P*_*pd*_ = 30 mW. The numerical detection RI range ∆*n* of the proposed structure varies linearly from *n* = 1 to *n* = 2.36, as shown by the red dashed line in Fig. 4a. The output normalized power variation ∆*P*_*num*_ of 0.78 is obtained numerically in Fig. 4a. The slope *m* of the linear variation is m = ∆*P*_*num*_/∆*n* = 0.57, where ∆*n* = 1.36 is the sensing range of the RI with a linear variance. As a result, the RI unit for the proposed SPR sensor is obtained by the equation: RIU = *δP*_*num*_/*m* = δ*P*_*pd*_/∆*P*_*pd ˙*_∆*n* = 4.53 × 10^−10^, where *δP*_*num*_ is the numerical resolution corresponding to the resolution of the chosen photodiode *δP*_*num*_ = ∆*P*_*num ˙*_ δ*P*_*pd*_/∆*P*_*pd*._

## Supplementary information

The electric field change with background refractive index.

The datasheet of Fig4b.

The datasheet of Fig4c.

The datasheet of Fig2.

The datasheet of Fig4a.

The datasheet of Fig5a.

The datasheet of Fig5b.

The datasheet of Fig3b.

The datasheet of Fig3b.
